# Pituitary extract supplementation enhances follicular survival and gonadotropin receptor expression in vitrified ovarian tissue of Aceh cattle, Indonesia

**DOI:** 10.14202/vetworld.2025.3607-3621

**Published:** 2025-11-29

**Authors:** Cut Intan Novita, Tongku Nizwan Siregar, Ni Wayan Kurniani Karja, Sri Wahyuni, Amalia Sutriana

**Affiliations:** 1 Animal Sciences Study Program, Faculty of Agriculture, Universitas Syiah Kuala, Banda Aceh, Indonesia; Graduate School of Mathematics and Applied Sciences, Universitas Syiah Kuala, Banda Aceh, Indonesia; 2 Laboratory of Reproduction, Faculty of Veterinary Medicine, Universitas Syiah Kuala, Banda Aceh, Indonesia; 3 Division of Reproduction and Obstetrics, School of Veterinary Medicine and Biomedical Sciences, IPB University, Bogor, Indonesia; 4 Laboratory of Anatomy, Faculty of Veterinary Medicine, Universitas Syiah Kuala, Banda Aceh, Indonesia; 5 Laboratory of Pharmacology, Faculty of Veterinary Medicine, Universitas Syiah Kuala, Banda Aceh, Indonesia

**Keywords:** Aceh cattle, caspase-3, cryopreservation, follicle-stimulating hormone receptor, luteinizing hormone receptor, pituitary extract, vitrification

## Abstract

**Background and Aim:**

Vitrification is a promising cryopreservation technique for conserving the genetic resources of Aceh cattle; however, it may induce cryoinjury and follicular apoptosis. Pituitary extract (PE), containing follicle-stimulating hormone (FSH) and luteinizing hormone (LH), can potentially mitigate apoptosis and maintain follicular viability. This study investigated the effect of bovine PE supplementation in vitrification media on the expression of caspase-3, FSH receptor (FSHR), and LH receptor (LHR) in vitrified ovarian tissue of Aceh cattle.

**Materials and Methods:**

A factorial, completely randomized design was employed using five vitrification media, PE at 0, 200, 400, and 600 μg/mL, and commercial bovine PE (Gibco, 300 μg/mL), combined with three vitrification durations (0, 7, and 14 days), each in triplicate. Post-vitrification ovarian tissue was evaluated by immunohistochemistry for caspase-3, FSHR, and LHR expression. The intensity scores (IS) were analyzed using the Kruskal–Wallis and Mann–Whitney U tests (p < 0.05).

**Results:**

PE supplementation significantly (p < 0.05) reduced caspase-3 expression across all follicular stages, indicating decreased apoptosis, whereas vitrification duration alone showed limited effects. The lowest caspase-3 IS (negative) occurred in the 600 μg/mL PE group after 7 days. Both PE concentration and vitrification duration significantly influenced FSHR and LHR expression (p < 0.05), with strong positive interactions between the two factors. FSHR and LHR expression increased progressively from primordial to antral follicles, suggesting preserved gonadotropin responsiveness. Optimal follicular preservation and receptor integrity were achieved at 600 μg/mL PE following 7 days of vitrification.

**Conclusion:**

Supplementation of vitrification media with 600 μg/mL bovine PE effectively suppresses follicular apoptosis while enhancing FSHR and LHR expression, maintaining ovarian functionality after vitrification. These results highlight PE as a low-cost, multi-hormonal alternative to single-gonadotropin supplements in cryopreservation media, supporting sustainable genetic conservation of Aceh cattle. Integrating locally derived PE into cryopreservation protocols could improve post-thaw follicular survival, reduce dependency on commercial reagents, and strengthen reproductive biotechnology for indigenous livestock conservation.

## INTRODUCTION

Aceh cattle represent one of Indonesia’s officially recognized indigenous genetic resources, as stipulated by the Ministry of Agriculture through Decree No. 2907/Kpts/OT.140/06/2011. Furthermore, the Indonesian National Standardization Agency formalized the national standard for Aceh cattle (SNI 7651.3:2013). Despite these designations, the breed’s population and genetic integrity have progressively declined due to uncontrolled crossbreeding with imported cattle breeds [1]. To counter this decline, a range of reproductive biotechnologies has been employed to enhance reproductive efficiency and population growth. These include estrus synchronization with prostaglandin F2α [2–4], the presynch–ovsynch protocol [5], artificial insemination [6], superovulation using pregnant mare serum gonadotropin and human chorionic gonadotropin [7, 8], and embryo transfer (ET) programs [7]. The success of ET largely depends on the availability of viable and high-quality embryos; however, limitations in both embryo yield and quality often restrict *in vivo* embryo production. Consequently, *in vitro* fertilization (IVF) has emerged as an alternative approach for generating transferable embryos [9, 10].

In IVF programs, oocytes are typically harvested from follicles within bovine ovaries collected as by-products from slaughterhouses. These ovaries, however, exhibit rapid degeneration even under cold storage, underscoring the necessity for efficient cryopreservation techniques. Vitrification, a rapid freezing process that prevents intracellular ice crystal formation, has shown great promise in preserving ovarian tissue quality. Supplementation of vitrification media with follicle-stimulating hormone (FSH) and luteinizing hormone (LH) has been demonstrated to reduce cryoinjury by supporting follicular growth, minimizing apoptosis, and maintaining post-thaw tissue functionality [11, 12]. Specifically, FSH and sphingosine-1-phosphate inhibit apoptosis and autophagy while enhancing follicular survival and angiogenesis [11], whereas LH promotes vascularization and receptor expression [12]. Recent evidence suggests that the combined physiological roles of FSH and LH can be effectively mimicked by pituitary extract (PE), a naturally derived, multi-hormonal preparation obtained from the pituitary glands of slaughtered animals.

The pituitary gland, usually discarded during meat processing, contains a spectrum of reproductive hormones capable of stimulating ovarian function. Previous research by Hafizuddin *et al*. [13] has shown that PE enhances reproductive performance by inducing superovulation in rats, improving ovarian response in goats [14], stimulating superovulation in Aceh cattle [15] and rabbits [16], and increasing the expression of ovarian hormones [17, 18]. The efficiency of PE supplementation in vitrification media can be assessed using histomorphological and molecular indicators, such as follicular morphology, follicular diameter, and the expression of caspase-3, FSH receptor (FSHR), and LH receptor (LHR). Caspase-3 acts as a critical apoptotic marker [19, 20], whereas FSHR and LHR reflect the sensitivity and functionality of ovarian cells in response to gonadotropins [21, 22].

Although vitrification has been established as an efficient cryopreservation technique for maintaining ovarian tissue integrity in various livestock species, its application in Aceh cattle remains underexplored. Most previous studies have primarily focused on optimizing cryoprotectant composition, cooling rates, and thawing procedures to reduce ice crystal formation and tissue injury. However, limited attention has been given to the biochemical and hormonal modulation of follicular survival during and after vitrification. The use of exogenous hormones such as FSH and LH has demonstrated promising anti-apoptotic and cytoprotective effects in bovine and ovine ovarian tissues. These hormones help preserve granulosa cell function, receptor sensitivity, and follicular morphology post-vitrification. Nevertheless, the application of *PE*, a multi-hormonal natural alternative containing both FSH and LH, along with other bioactive factors, has not yet been investigated as a supplement in vitrification media for Aceh cattle.

Furthermore, the majority of existing studies evaluate ovarian tissue viability through morphological assessment alone, without integrating molecular markers of apoptosis and follicular activity. The expression profiles of caspase-3, FSHR, and LHR provide critical insight into the underlying cellular mechanisms that determine follicular survival, yet these parameters have not been comprehensively examined in the context of PE-supplemented vitrification. In addition, the dose-dependent and temporal responses of ovarian follicles to PE supplementation during short-term storage remain unclear. This gap in knowledge limits the development of an optimized cryopreservation strategy tailored for indigenous cattle breeds like the Aceh, whose genetic resources are threatened by crossbreeding and population decline.

This study was designed to investigate the effect of bovine PE supplementation in vitrification media on the post-vitrification quality of Aceh cattle ovarian tissue. Specifically, it aimed to (1) evaluate the influence of varying PE concentrations and vitrification durations on the expression of caspase-3, FSHR, and LHR as key indicators of apoptosis and hormonal responsiveness; (2) determine the optimal PE concentration and storage period that best preserve follicular structure and functionality; and (3) explore the potential of PE as a low-cost, multi-hormonal alternative to commercial gonadotropins for use in cryopreservation protocols. By integrating immunohistochemical and quantitative analyses, this study provides mechanistic evidence supporting the use of PE in improving ovarian tissue viability and receptor integrity, thereby contributing to sustainable reproductive biotechnology and genetic conservation of Indonesia’s indigenous Aceh cattle.

## MATERIALS AND METHODS

### Ethical approval

This study did not involve any live animal experimentation. Ovarian tissues and pituitary glands were obtained post-mortem from routinely slaughtered Aceh cattle at the Lambaro Municipal Abattoir (Aceh Besar District, Indonesia), following standard meat-processing procedures. Therefore, in accordance with the institutional and national regulations, the Animal Ethics Committee of the Faculty of Veterinary Medicine, Universitas Syiah Kuala, waived the requirement for ethical review as no procedures were performed on live animals.

All procedures involving biological samples strictly adhered to:

- The Institutional Guidelines for the Use of Animal-derived Materials in Research (Biosafety Level 2 standards).
- The Indonesian Ministry of Agriculture regulations governing the handling of animal by-products.
- OIE (World Organization for Animal Health) animal welfare principles, ensuring that samples were collected without causing additional distress or harm to the animals.

Post-collection handling, transport, vitrification, and laboratory processing of ovarian tissues were conducted under controlled conditions to maintain sample integrity while complying with institutional biosafety and biowaste disposal protocols.

### Study period and location

The study was conducted from November 2023 to February 2025.

- Sample collection sites: Ovaries and pituitary glands were obtained from the Lambaro Slaughterhouse, Aceh Besar District, Aceh Province, Indonesia.
- Experimental procedures: Vitrification and media preparation were carried out at the Laboratory of Animal Breeding and Reproduction, Faculty of Agriculture, Universitas Syiah Kuala.
- Immunohistochemical analyses: Performed at the Anatomy Laboratory, Faculty of Veterinary Medicine, Universitas Syiah Kuala.

### Experimental design

A factorial completely randomized design was employed to evaluate the effects of bovine PE concentration and vitrification duration.

### Treatment factors

- PE concentrations: 0, 200, 400, and 600 µg/mL.
- Positive control: Commercial bovine PE (BPE; Gibco, UK) at 300 µg/mL [23].
- Vitrification durations: 0, 7, and 14 days. Each treatment combination was replicated 3 times, resulting in 15 combinations and a total of 45 ovarian samples.

### Pituitary extraction

Twelve pituitary glands were collected from adult male Aceh cattle (3–5 years old) slaughtered at a local abattoir in Banda Aceh. Animals were selected based on the criteria of the Minister of Agriculture Decree No. 2907/Kpts/OT.140/6/2011, ensuring a normal body condition score (3–3.5) and absence of pathological lesions in the pituitary region.

Extraction followed the method of Sutiyono *et al*. [24] with modifications:

1. Glands were soaked in 90% ethanol for 16 h, with ethanol replaced every 4 h to dehydrate tissues.
2. Dried glands were air-dried and ground into fine powder.
3. The powder was mixed with 0.9% sodium chloride (NaCl) solution (10 mL/g) and centrifuged at 100 × *g* for 10 min.
4. The supernatant served as PE.

### Hormonal analysis

Each 100 µL of PE contained 242.99 mIU/mL FSH and 32.09 mIU/mL LH, while commercial BPE (Gibco) contained 548.65 mIU/mL FSH and 87.72 mIU/mL LH.

### Ovary collection

Ovaries were collected from reproductively active, healthy female Aceh cattle (3–5 years old, previously calved) immediately post-slaughter. Only right ovaries without corpus luteum, weighing 4–8 g, and free of abnormalities were included.

Samples were placed in 0.9% physiological saline containing 100 IU/mL penicillin and 0.1 g/mL streptomycin, transported in thermos containers at 35°C, with continuous monitoring using a thermometer. The time between slaughter and processing did not exceed 60 min [25].

### Ovary vitrification procedure

The vitrification protocol was adapted from Djuwita *et al*. [25] with modifications:

- No FSH or NCS supplementation was used.
- Ovaries were exposed to vitrification medium for 15 min (vs. 5 min in the original method).

### Stepwise dehydration

1. Ovaries were sequentially equilibrated in phosphate-buffered saline (PBS; pH 7.4) + sucrose (0.25 and 0.5 M; 5 min each).
2. Transferred to vitrification media containing PBS, 0.5 M sucrose, 30% (v/v) ethylene glycol, and PE according to treatment groups (V0–V4).
3. Dehydration was conducted under 25°C laminar airflow for 25 min.
4. Samples were exposed to liquid nitrogen vapor (−140°C) for 10 s, then fully immersed in liquid nitrogen (−196°C), achieving a cooling rate of 20,000–50,000°C/min.

### Warming and recovery

Ovaries were warmed in a 35°C water bath, then washed through graded PBS–sucrose solutions (5 min each) and fixed in 10% neutral buffered formalin (NBF) for histological preparation.

### Histology and immunohistochemistry (IHC)

Post-warming, ovaries were processed using standard histological procedures:

- Fixation and embedding: Fixed in 10% NBF, embedded in paraffin, and sectioned at 4 μm thickness.
- Staining: Sections were stained with hematoxylin and eosin for morphological evaluation and with IHC for protein expression analysis.

### Primary antibodies used

- Anti-Caspase-3 (Bioss Inc., Woburn, Massachusetts, USA, catalog no. BS-0081R; lot ZF4360222).
- Anti-FSHR (Biossusa Inc., catalog no. BS-0895R, lot ZF4360223).
- Anti-LHR (Biossusa Inc., catalog no. BS-6431R, lot ZF4360229A).

### IHC procedure

- Antigen retrieval in citrate buffer (pH 6.0) at 95°C for 15 min.
- Blocking with 3% hydrogen peroxide and 5% bovine serum albumin.
- Primary antibody incubation (1:200) overnight at 4°C.
- Detection with horseradish peroxidase-conjugated secondary antibody and visualization using 3,3´-diaminobenzidine substrate, followed by hematoxylin counterstaining.
- Immunoreactivity scoring: 0 (negative), 1 (weak), 2 (moderate), 3 (strong) [10].

### Blinded evaluation

Each slide was coded and evaluated independently by three observers (one expert pathologist and two trained researchers).

### Statistical analysis

Expression data for caspase-3, FSHR, and LHR were descriptively summarized and illustrated using histological images. Statistical analyses were performed using the Statistical Package for the Social Sciences version 25.0 (IBM Corp., NY, USA).

- Group differences were evaluated using the Kruskal–Wallis test.
- Significant results were followed by Mann–Whitney U tests with Bonferroni correction to adjust for multiple comparisons.
- A p < 0.05 was considered statistically significant.

## RESULTS

### Caspase-3 expression

**Figure 1:**
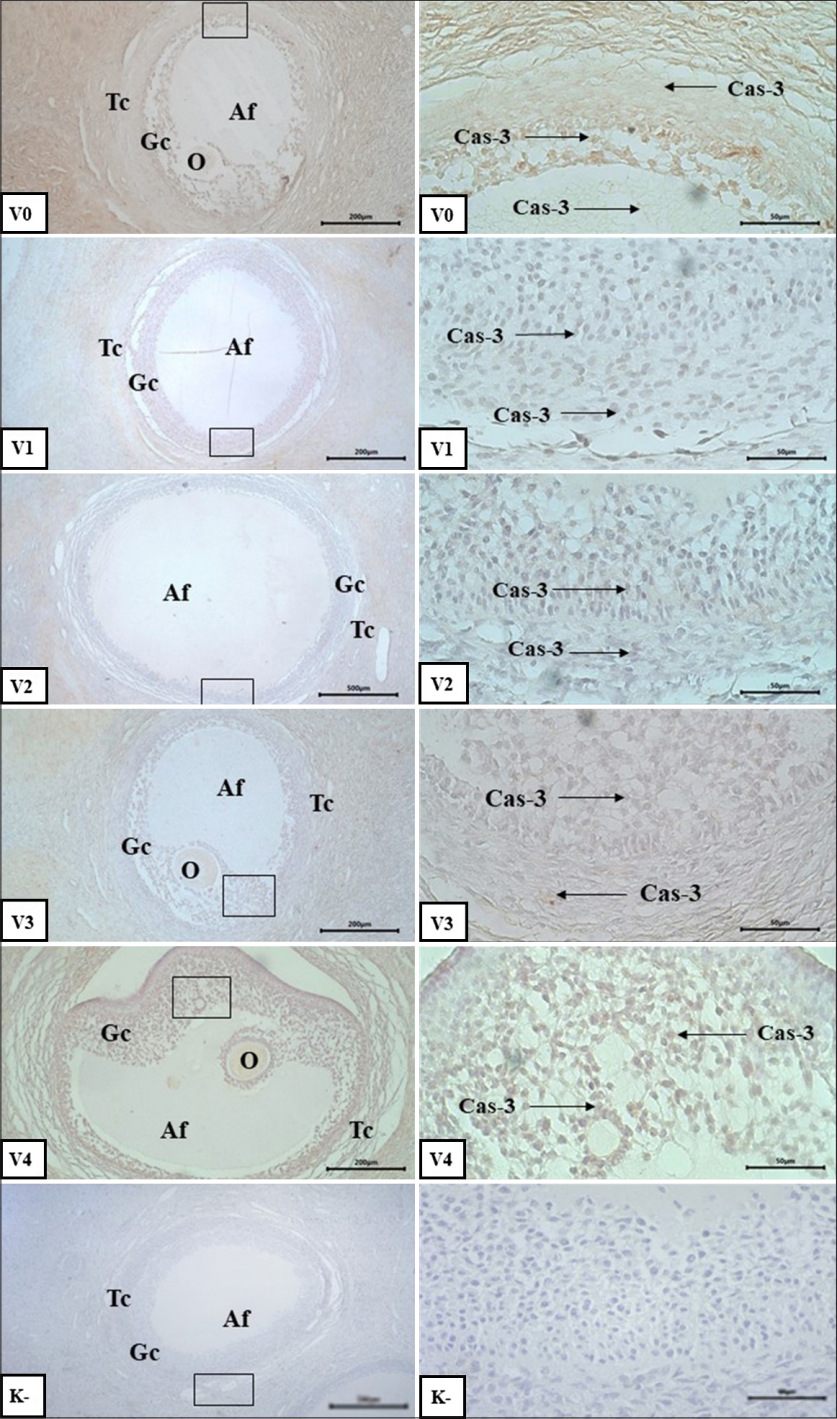
Caspase-3 expression in antral follicles on day 14 vitrification; V0: bovine pituitary extract (PE) 0 μg/mL; V1: bovine PE 200 μg/mL; V2: bovine PE 400 μg/mL; V3: bovine PE 600 μg/mL; V4: bovine PE (Gibco) 300 μg/mL; K-: negative control; oocyte (o); theca cell (Tc); granulosa cell (Gc); expression of caspase-3 (cas-3, arrow); immunohistochemical staining; bar scale 200 μm for V0, V1, V2, V3, and V4 (left column); bar scale 50 μm for V0 and V1. V2. V3 and V4 (right column/inset).

Post-vitrification examination of Aceh cattle ovarian tissue revealed caspase-3 expression in granulosa cells and follicular fluid across nearly all vitrification media treatments (Figure 1). The expression intensity was evaluated using the intensity score (IS) method, where higher IS values indicate stronger expression and a greater degree of apoptosis.

Caspase-3, a hallmark apoptotic marker, was detected at low-to-moderate intensity, suggesting that PE supplementation effectively reduced apoptotic activity in vitrified ovarian tissue. Statistical analysis confirmed that PE supplementation significantly influenced (p < 0.05) caspase-3 expression at all follicular developmental stages, while vitrification duration alone showed no significant effect (p > 0.05). However, a significant interaction between PE concentration and vitrification duration was observed.

Caspase-3 expression scores ranged from negative (score 0) to moderate (score 2), indicating varying levels of apoptosis depending on treatment (Table 1). The lowest expression was typically recorded in the 600 µg/mL PE group, highlighting its protective effect against cryo-induced apoptosis.

**Table 1:** Median (IQR) intensity score of caspase-3 expression in Aceh cattle ovarian follicles due to the combination of bovine pituitary extract supplementation and vitrification duration treatments.

Bovine PE concentration	Vitrification duration			Median (IQR)

	0 day	7 days	14 days	
Primordial follicle				
V0, PE 0 μg/mL	1.53 ± 0.47^bcd^	1.70 ± 0.60^cd^	1.90 ± 0.17^d^	1.70 (0.15)^b^
V1, PE 200 μg/mL	1.93 ± 0. 92^d^	1.09 ± 0.34^abcd^	1.09 ± 0.34^abcd^	1.09 (0.00)^b^
V2, PE 400 μg/mL	0.63 ± 1.09^ab^	0.66 ± 0.57^ab^	0.70 ± 0.26^ab^	0.66 (0.41)^a^
V3, PE 600 μg/mL	0.46 ± 0.05^a^	0,36 ± 0,28^a^	1.13 ± 0.23^abcd^	0.36 (0.11)^a^
V4, PE (Gibco) 300 μg/mL	0.90 ± 0.43^a^	1.26 ± 0.37^abcd^	1.73 ± 0.25^cd^	1.26 (0.09)^b^
Median (IQR)	0.90 (0.29)^a^	1.09 (0.09)^a^	1.13 (0.03)^a^	
Primary follicle				
V0, PE 0 μg/mL	1.23 ± 0.40^ab^	1.89 ± 0.57^cd^	2.67 ± 0.58^d^	1.89 (0.09)^c^
V1, PE 200 μg/mL	1.00 ± 1.32^ab^	0.53 ± 0.6^a^	1.00 ± 0.00^ab^	1.00 (0.66)^ab^
V2, PE 400 μg/mL	0.60 ± 0.87^a^	0.77 ± 0.68^a^	0.63 ± 0.35^a^	0.63 (0.26)^a^
V3, PE 600 μg/mL	0.37 ± 0.23^a^	0.33 ± 032^a^	1.10 ± 0.1^ab^	0.37 (0.11)^a^
V4, PE (Gibco) 300 μg/mL	1.25 ± 0.22^ab^	1.00 ± 0.00^ab^	1.80 ± 0.17^bc^	1.25 (0.11)^b^
Median (±IQR)	1.00 (0.64)^a^	0.77 (0.28)^a^	1.10 (0.25)^b^	
Secondary follicle				
V0, PE 0 μg/mL	1.17 ± 029^cd^	2.17 ± 0.29^e^	1.83 ± 0.29^de^	1.83 (0.00)^d^
V1, PE 200 μg/mL	1.25 ± 0.00^cd^	0.86 ± 0.50^abc^	1.00 ± 0.00^bc^	1.00 (0.25)^ab^
V2, PE 400 μg/mL	1.17 ± 0.76^cd^	1.00 ± 0.00^bc^	0.20 ± 0.34^a^	1.00 (0.38)^ab^
V3, PE 600 μg/mL	0.33 ± 0.29^ab^	0.17 ± 0.29^a^	1.10 ± 0.17^c^	0.33 (0.06)^a^
V4, PE (Gibco) 300 μg/mL	2.10 ± 0.17^e^	1.25 ± 0.25^cd^	1.00 ± 0.87^bc^	1.25 (0.35)^c^
Median (±IQR)	1.17 (0.12)^a^	1.00 (0.04)^a^	1.00 (0.17)^a^	
Antral follicle				
V0, PE 0 μg/mL	1.50 ± 0.50^abc^	1.67 ± 0.58^abc^	2.00 ± 0.10^bc^	1.67 (0.24)^b^
V1, PE 200 μg/mL	2.33 ± 1.15^c^	1.32 ± 0.39^ab^	1.37 ± 0.32^ab^	1.37 (0.41)^ab^
V2, PE 400 μg/mL	1.38 ± 1.04^ab^	0.83 ± 0.14^a^	1.10 ± 0.17^ab^	1.10 (0.45)^a^
V3, PE 600 μg/mL	0.71 ± 0.06^a^	1.38 ± 0.19^ab^	1.25 ± 0.25^ab^	1.25 (0.09)^a^
V4, PE (Gibco) 300 μg/mL	1.45 ± 0.18^abc^	1.40 ± 0.36^ab^	1.90 ± 0.17^bc^	1.45 (0.09)^ab^
Median (±IQR)	1.45 (0.86)^a^	1.38 (0.20)^a^	1.37 (0.08)^a^	

Different superscripts within the same row and column indicate a significant difference (p < 0.05)*.* IQR = Interquartile range, PE = Pituitary extract.

### FSHR expression

The expression of the FSHR increased progressively with follicular maturation, indicating preserved responsiveness of ovarian follicles to gonadotropins. PE supplementation significantly enhanced FSHR expression, demonstrating improved follicular viability and hormonal sensitivity following vitrification.

**Figure 2:**
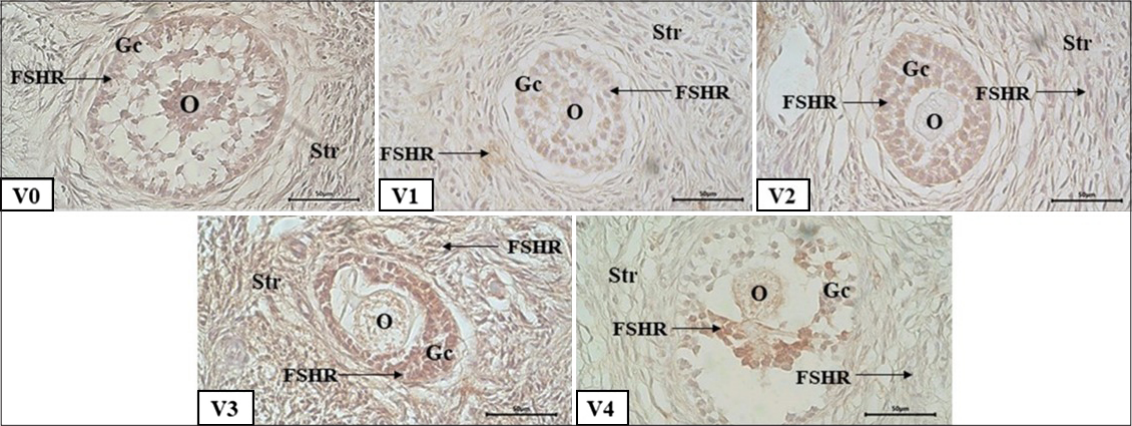
Follicle-stimulating hormone receptor (FSHR) expression in secondary follicles on day 14 of vitrification; V0: bovine pituitary extract (PE) 0 μg/mL; V1: bovine PE 200 μg/mL; V2: bovine PE 400 μg/mL; V3: bovine PE 600 μg/mL; V4: bovine PE (Gibco) 300 μg/mL; oocyte (O); theca cell (Tc); granulosa cell (Gc); FSHR expression (arrow); immunohistochemical staining; bar scale 50 μm.

Both PE concentration and vitrification duration had significant effects (p < 0.05) on FSHR expression intensity, with a notable interaction between the two factors (Table 2). FSHR was localized primarily in granulosa cells across all follicular stages, primordial, primary, secondary, and antral, with expression intensity rising with follicular development (Figure 2). These results suggest that optimal PE concentrations can sustain receptor-mediated follicular function during cryopreservation.

**Table 2:** Median (IQR) intensity score of FSHR expression in Aceh cattle ovarian follicles after treatment with a combination of bovine pituitary extract supplementation and vitrification.

Bovine PE concentration	Vitrification duration			Median (IQR)

	0 day	7 days	14 days	
Primordial follicle				
V0, PE 0 μg/mL	0.81 ± 0.25^bcd^	0.86 ± 0.12^cde^	0.43 ± 0.05^abc^	0.81 (0.97)^a^
V1, PE 200 μg/mL	1.26 ± 0.23^ef^	1.20 ± 0.36^def^	1.37 ± 0.23^f^	1.26 (0.06)^b^
V2, PE 400 μg/mL	1.26 ± 0.55^ef^	1.78 ± 0.10^g^	1.90 ± 0.10^g^	1.78 (0.22)^c^
V3, PE 600 μg/mL	0.65 ± 0.13^abc^	0.41 ± 0.10^ab^	2.08 ± 0.18^g^	0.65 (0.04)^a^
V4, PE (Gibco) 300 μg/mL	0.28 ± 0.10^a^	0.70 ± 0.17^abc^	2.73 ± 0.30^h^	0.70 (0.10)^a^
Median (IQR)	0.81 (0.12)^a^	0.86 (0.26)^a^	1.90 (0.13)^b^	
Primary follicle				
V0, PE 0 μg/mL	0.90 ± 0.10^abcd^	0.95 ± 0.05^abcde^	0.53 ± 0.11^ab^	0.90 (0.03)^a^
V1, PE 200 μg/mL	1.00 ± 0.50^abcde^	1.06 ± 0.72^bcde^	1.50 ± 0.43^def^	1.06 (0.14)^b^
V2, PE 400 μg/mL	1.20 ± 0.34^cde^	0.68 ± 0.16^abc^	1.53 ± 0.11^ef^	1.20 (0.11)^b^
V3, PE 600 μg/mL	0.76 ± 0.25^abc^	0.36 ± 0.05^a^	2.05 ± 0.22^fg^	0.76 (0.10)^a^
V4, PE (Gibco) 300 μg/mL	0.55 ± 0.13^ab^	0.85 ± 0.13^abc^	2.36 ± 0.56^g^	0.85 (0.21)^a^
Median (IQR)	0.90 (0.21)^a^	0.85 (0.11)^a^	1.53 (0.32)^b^	
Folikel Sekunder				
V0, PE 0 μg/mL	1.00 ± 0.00^abcd^	0.83 ± 0.15^ab^	1.78 ± 0.10^e^	1.00 (0.07)^a^
V1, PE 200 μg/mL	1.50 ± 0.25^cde^	1.13 ± 0.76^bcd^	1.40 ± 0.40^bcde^	1.40 (0.25)^a^
V2, PE 400 μg/mL	0.93 ± 0.11^abc^	1.40 ± 0.36^bcde^	1.56 ± 0.51^cde^	1.40 (0.20)^a^
V3, PE 600 μg/mL	1.03 ± 0.05^abcd^	0.45 ± 0.39^a^	2.73 ± 0.25^f^	1.03 (0.17)^a^
V4, PE (Gibco) 300 μg/mL	1.83 ± 0.15^e^	1.93 ± 0.05^e^	1.58 ± 0.38^de^	1.83 (0.16)^b^
Median (IQR)	1.03 (0.10)^a^	1.13 (0.24)^a^	1.58 (0.15)^b^	
Folikel Antral				
V0, PE 0 μg/mL	1.50 ± 0.10^abc^	1.00 ± 0.00^a^	1.93 ± 0.05^cd^	1.50 (0.05)^a^
V1, PE 200 μg/mL	1.55 ± 0.48^abc^	1.78 ± 0.60^bc^	1.48 ± 0.20^abc^	1.55 (0.20)^a^
V2, PE 400 μg/mL	1.26 ± 0.30^ab^	0.96 ± 0.05^a^	2.41 ± 0.52^def^	1.26 (0.23)^a^
V3, PE 600 μg/mL	1.00 ± 0.00^a^	1.00 ± 0.00^a^	2.63 ± 0.28^f^	1.00 (0.14)^a^
V4, PE (Gibco) 300 μg/mL	2.03 ± 0.41^cde^	1.26 ± 0.25^ab^	2.56 ± 0.51^ef^	2.03 (0.13)^b^
Median (IQR)	1.31 (0.44)^b^	1.00 (0.25)^a^	2.41 (0.31)^c^	

Different superscripts within the same row and column indicate a significant difference (p < 0.05). IQR = Interquartile range, PE = Pituitary extract, FSHR = Follicle-stimulating hormone receptor.

### LHR expression

**Figure 3:**
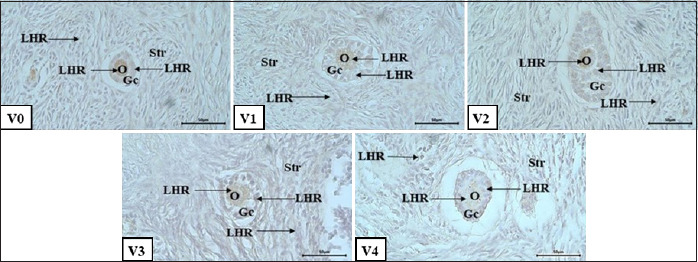
Luteinizing hormone receptor (LHR) expression in secondary follicles on day 7 of vitrification; V0: bovine pituitary extract (PE) 0 μg/mL; V1: bovine PE 200 μg/mL; V2: bovine PE 400 μg/mL; V3: bovine PE 600 μg/mL; V4: bovine PE (Gibco) 300 μg/ml; oocyte (o); theca cell (Tc); granulosa cell (Gc); LHR expression (LHR, arrow); immunohistochemical staining; bar scale 50 μm.

The LHR was consistently expressed at all follicular stages in vitrified Aceh cattle ovaries. Immunohistochemical visualization using the avidin–biotin complex method revealed LHR expression as brown cytoplasmic staining within granulosa cells (Figure 3).

Quantitative evaluation using the IS method (Table 3) showed that both PE supplementation and vitrification duration significantly influenced (p < 0.05) LHR expression across most follicular stages. However, vitrification duration alone did not significantly affect LHR expression in secondary follicles (p > 0.05), although an interaction between the two factors remained evident.

**Table 3:** Median (IQR) intensity score of LHR expression in Aceh cattle ovarian follicles due to the combination of bovine pituitary extract supplementation and vitrification duration treatments.

Bovine PE concentration	Vitrification duration			Median (IQR)

	0 day	7 days	14 days	
Primordial follicle				
V0, PE 0 μg/mL	0.86 ± 0.66^bcd^	0.86 ± 0.40^bcd^	0.76 ± 0,40^bcd^	0.86 (0.13)^b^
V1, PE 200 μg/mL	0.00 ± 0,00^a^	0.48 ± 0.38^ab^	0.56 ± 0.28^abc^	0.48 (0.19)^a^
V2, PE 400 μg/mL	1.21 ± 0.20^cde^	0.80 ± 0.32^bcd^	0.50 ± 0.20^ab^	0.80 (0.06)^b^
V3, PE 600 μg/mL	1.13 ± 0.15^bcde^	0.96 ± 0.05^bcd^	1.11 ± 0.12^bcde^	1.11 (0.05)^bc^
V4, PE (Gibco) 300 μg/mL	1.73 ± 0.64^e^	1.01 ± 0.07^bcd^	1.23 ± 0.20^de^	1.23 (0.28)^c^
Median (IQR)	1.13 (0.49)^b^	0.86 (0.31)^a^	0.76 (0.08)^a^	
Primary follicle				
V0, PE 0 μg/mL	0.60 ± 0.60^abcd^	1.10 ± 0.52^def^	0.93 ± 0.11^cdef^	0.93 (0.24)^bc^
V1, PE 200 μg/mL	0.06 ± 0.11^a^	0.26 ± 0.30^ab^	0.36 ± 0.15^abc^	0.26 (0.09)^a^
V2, PE 400 μg/mL	0.85 ± 0.13^bcdef^	0.86 ± 0.15^bcdef^	0.50 ± 0.10^abcd^	0.85 (0.02)^b^
V3, PE 600 μg/mL	1.21 ± 0.40^ef^	0.73 ± 0.25^bcde^	1.46 ± 0.45^fg^	1.21 (0.10)^c^
V4, PE (Gibco) 300 μg/mL	1.86 ± 0.32^g^	1.23 ± 0.25^ef^	1.40 ± 0.36^fg^	1.40 (0.05)^d^
Median (IQR)	0.85 (0.27)^a^	0.86 (0.05)^a^	0.93 (0.25)^a^	
Secondary follicle				
V0, PE 0 μg/mL	1.00 ± 1.00^c^	1.16 ± 0.28^cd^	1.00 ± 0.20^c^	1.00 (0.40)^a^
V1, PE 200 μg/mL	0.93 ± 0.49^bc^	0.20 ± 0.34^ab^	0.70 ± 0.52^abc^	0.70 (0.09)^a^
V2, PE 400 μg/mL	1.00 ± 0.00^c^	1.00 ± 0.00^c^	0.93 ± 0.11^bc^	1.00 (0.05)^a^
V3, PE 600 μg/mL	0.60 ± 0.52^abc^	1.80 ± 0.17^de^	1.00 ± 0.00^c^	1.00 (0.26)^a^
V4, PE (Gibco) 300 μg/mL	1.93 ± 0.60^e^	1.13 ± 0.15^cd^	0.00 ± 0.00^a^	1.13 (0.30)^a^
Median (IQR)	1.00 (0.11)^a^	1.13 (0.17)^a^	0.93 (0.20)^a^	
Antral follicle				
V0, PE 0 μg/mL	0.87 ± 0.41^a^	1.08 ± 0.14^abc^	0.93 ± 0.11^ab^	0.93 (0.15)^a^
V1, PE 200 μg/mL	1.16 ± 0.15^abcd^	0.93 ± 0.57^ab^	1.43 ± 0.05^abcdf^	1.16 (0.26)^ab^
V2, PE 400 μg/mL	1.06 ± 0.11^abc^	1.53 ± 0.41^bcdef^	1.98 ± 0.28^fg^	1.53 (0.15)^b^
V3, PE 600 μg/mL	1.73 ± 0.37^def^	1.33 ± 0,57^abcde^	1.16 ± 0,28^abcde^	1.33 (0.14)^b^
V4, PE (Gibco) 300 μg/mL	1.90 ± 0.17^efg^	1.63 ± 0.11^cdef^	2.33 ± 0.35^g^	1.90 (0.12)^c^
Median (IQR)	1.16 (0.22)^a^	1.33 (0.43)^ab^	1.43 (0.17)^c^	

Different superscripts within the same row and column indicate a significant difference (p < 0.05)*.* IQR = Interquartile range, PE = Pituitary extract, LH = Luteinizing hormone.

LHR expression intensity varied from negative to moderate, depending on PE concentration and storage duration. Expression patterns differed among follicular types, with fluctuating levels in primordial and primary follicles, more stable expression in secondary follicles, and the strongest expression observed in antral follicles.

These findings indicate that PE supplementation helps maintain follicular architecture, reduces apoptotic activity, and preserves receptor-mediated hormonal responsiveness in post-vitrification ovarian tissue of Aceh cattle.

## DISCUSSION

### Caspase-3 expression and apoptotic pathways

#### Functional role of caspase-3 in follicular apoptosis

Caspase-3 is a key enzyme in the apoptotic pathway and is often referred to as the executioner caspase due to its direct role in executing cell death. Its expression increases when cells experience stress or damage, including during cryopreservation procedures such as vitrification. Apoptosis in vitrified ovaries can lead to the depletion of the PF reserve. Caspase-3 is the main executor of apoptosis in ovarian follicles, particularly during follicular atresia [26, 27]. Caspase-3 activity is significantly higher in granulosa cells of atretic follicles than in intact ones, indicating its role in follicular degeneration [28]. Caspase-3 expression intensity in Aceh cattle ovarian tissue post-vitrification was visualized by IHC staining.

#### Caspase-3 expression across follicular stages and treatments

In primordial follicles, the lowest (negative) expression of caspase-3 was observed in the 600 μg/mL PE group after 7 days, whereas the highest (moderate) expression was found in the 200 μg/mL PE group at day 0. Caspase-3 expression scores ranged from negative (score 0) to moderate (score 2). The variation in expression across treatments suggests that higher PE concentrations tend to reduce caspase-3 expression. Without PE or in the PE 300 μg/mL-positive control group, caspase-3 expression increased with longer vitrification periods. In contrast, treatment with 200–400 μg/mL PE tended to stabilize or reduce the expression of apoptosis. The 600 μg/mL PE treatment showed the best protective effect, with negative to weak expression.

In primary follicles, caspase-3 expression was also significantly affected by both factors (p < 0.05). Apoptosis increased with the duration of vitrification in the untreated group, whereas the 200–400 μg/mL PE groups showed consistently low expression. The 600 μg/mL PE group suppressed caspase-3 expression maximally at days 0 and 7 but slightly increased at day 14.

Caspase-3 expression in secondary follicles followed a similar pattern. PE supplementation had a significant effect (p < 0.05), but the vitrification duration did not. The best combination was 600 μg/mL PE for 7 days (negative expression), and the worst was 0 μg/mL PE for 7 days. Expression was more variable and generally higher in the untreated group than in the supplemented group.

Caspase-3 expression in antral follicles ranged from weak to moderate (Figure 1). PE supplementation had a significant effect, but vitrification duration did not. The best outcome was achieved in the 600 μg/mL PE group on day 0. Overall, PE, especially at 600 μg/mL, suppressed caspase-3 expression across all follicular stages. These findings suggest that the FSH and LH contents in PE may reduce apoptosis during vitrification. This reduction in granulosa cell apoptosis is likely mediated by the activation of the Protein Kinase A (PKA), Protein Kinase B (Akt), p38 mitogen-activated protein kinase, and extracellular signal-regulated kinase 1 and 2 (ERK1/2) pathways [29] and the inhibition of mitophagy under oxidative stress [30]. LH may inhibit apoptosis through activation of the transforming growth factor-beta (TGF-β) pathway and upregulation of forkhead box L2 (Foxl2), which suppresses caspase-3 activation [31].

### FSHR expression and follicular responsiveness

#### Significance of FSHR in follicular function

FSHR expression is an important indicator for assessing follicular activity and function. The purpose of examining FSHR expression was to evaluate whether PE-supplemented ovarian follicles could retain FSHR expression, which is essential for subsequent *in vitro* embryo production applications.

### FSHR expression patterns across follicular stages

FSHR was detected in granulosa cells of all follicle types, from primordial to antral, with increasing expression intensity with follicular maturation (Table 2). Previous studies by Chen *et al*. [32] and Saraiva *et al*. [33] have also reported a significant increase in FSHR from preantral to antral follicles. Statistically, both PE supplementation and vitrification duration significantly affected (p < 0.05) FSHR immunoreactivity scores across all follicle types, with a significant interaction between factors.

In primordial follicles, FSHR expression was relatively weak but increased significantly with higher PE doses and longer vitrification. This early activation may be due to the stimulation of FSHR expression by FSH in PE even in dormant follicles. Vitrification itself can induce sublethal stress that activates the phosphoinositide 3-kinase (PI3K)-Akt and mammalian target of rapamycin signaling pathways, which are known to trigger the activation of early primordial follicles [34, 35]. This aligns with Bhartiya *et al*. [36], who stated that although FSHR is typically low at this stage, it can be upregulated exogenously.

FSHR expression was stronger in primary follicles than in primordial follicles and significantly increased with PE supplementation, especially in the 14-day vitrification group. Exogenous hormone stimulation accelerated the follicular response to growth signals. According to Tisdall *et al*. [37], FSH is essential for transitioning from primary to secondary follicles by increasing receptor gene expression and promoting GC proliferation.

FSHR expression in secondary follicles was higher than that in primary follicles. PE supplementation further increased expression, especially in the 14-day vitrification group, indicating that prolonged exposure to exogenous FSH may accelerate follicular maturation. This is supported by Zhang *et al*. [38], who highlighted the role of FSH in supporting GC growth and differentiation during the secondary follicle stage.

The antral follicles showed the highest FSHR expression among all follicle types (Figure 2). This expression was further enhanced by the combination of PE dose and 14-day vitrification. The response reflects the dominant role of FSH in facilitating antral follicle development, particularly in stimulating granulosa cells to synthesize estrogen [39]. However, excessive FSH stimulation may increase the risk of ovarian overstimulation and endocrine disruption [40, 41].

### LHR expression and follicular maturation

#### Role of LHR in follicular development

The LHR plays a crucial role in follicular maturation. LH expression is an important marker for assessing follicle readiness and responsiveness to hormonal stimulation. LH plays a central role in oocyte maturation, ovulation, and luteinization, and it is also involved in signal transduction pathways that regulate follicular survival and growth [42, 43]. Anti-apoptotic roles of FSH and LH act synergistically to support follicular viability.

### LHR expression in primordial and primary follicles

In the group with no PE supplementation (0 μg/mL), LHR expression remained stable across different vitrification durations (no significant difference), consistently showing weak expression. At 200 μg/mL PE, LHR expression was absent in primordial follicles on day 0, but slightly increased on days 7 and 14. Conversely, the 400 μg/mL PE group showed the highest expression on day 0 (weak expression), followed by a gradual decrease on days 7 and 14. The 600 μg/mL PE group exhibited relatively stable and weak expression throughout the study. Meanwhile, BPE 300 μg/mL on day 0 induced the strongest LHR expression (moderate category), which then declined to weak expression on days 7 and 14.

LHR expression in primordial follicles exhibited inconsistent responses that were not directly correlated with PE concentration or vitrification duration. For example, the 200 μg/mL group had lower expression scores than the untreated group. The positive effects of PE supplementation were observed only at 400–600 μg/mL concentrations and in the BPE 300 μg/mL group. This suggests that 400–600 μg/mL PE may enhance LHR expression in primordial follicles after vitrification. Interestingly, LHR was still expressed despite primordial follicles being physiologically dormant. This result supports the findings of Giroto *et al*. [44], who reported LHR protein expression in oogonia and primordial follicles in bovine fetal ovaries.

LHR expression was detected in primary follicles in all groups receiving PE supplementation, with intensities ranging from weak to moderate (Figure 3). The intensity of LHR expression varied depending on the PE concentration and the vitrification duration. Expression remained weak and fluctuated across storage times in the control group (no PE). The 200 μg/mL PE group showed no LHR expression, whereas the 400 μg/mL group had stable weak expression throughout the study period. The 600 μg/mL group showed fluctuating expression: it declined on day 7 but rose again on day 14 although still within the weak category. The bovine PE 300 μg/mL group showed the highest LHR expression on day 0 (moderate), which gradually declined to weak on days 7 and 14.

Overall, increased LHR expression was particularly evident on day 14 in the 600 μg/mL PE and BPE groups, suggesting that early LHR expression may play a role in maintaining cell viability after cryopreservation. This aligns with a previous study by Yamashita *et al*. [45] indicating that early LHR expression supports GC survival and proliferation during early follicular development. A noteworthy phenomenon was observed: in primordial and primary follicles, the absence of PE supplementation sometimes resulted in better LHR expression than the 200 μg/mL group. The lack of expression at this concentration suggests that it may be insufficient to induce LHR presence. On the other hand, the absence of LHR might be a mechanism that prevents premature follicular activation during vitrification. In the non-supplemented group, however, LHR expression occurred, possibly reflecting a natural follicular adaptation or response to the vitrification environment. At 600 μg/mL PE and BPE 300 μg/mL with 7-day vitrification, the average LHR expression scores declined, suggesting that day 7 may be an optimal period during which lower LHR expression helps maintain follicular quiescence or prevent premature activation. On day 14, however, LHR expression increased significantly in these groups, indicating possible LHR upregulation due to high concentration and prolonged exposure.

### LHR expression in secondary and antral follicles

LHR expression in primary follicles is a key marker of follicular maturity and LH responsiveness. Phoophitphong *et al*. [46] reported the presence of LHR at the primary follicle stage, particularly in granulosa and theca cells, indicating its role in early follicular development. In contrast, Yung *et al*. [47] did not detect LHR in primordial or primary follicles through protein staining; LHR expression only appeared in small antral follicles. In this study, the presence of LHR in primary follicles suggests that early follicular activation or adaptation to post-vitrification conditions is occurring. Bovine PE contains various hormones, including FSH and LH, that may directly or indirectly stimulate LHR expression in primary follicle granulosa and theca cells.

The secondary follicles displayed relatively stable LHR expression across all treatment groups, although the mean values decreased slightly by day 14. The 600 μg/mL PE group exhibited the highest expression on day 7, indicating a peak stimulatory effect at the mid-vitrification stage. By contrast, expression dropped to zero in the BPE 300 μg/mL group by day 14, possibly due to reduced compatibility of the commercial preparation with the follicular physiology of Aceh cattle.

Secondary follicles are characterized by five or more layers of granulosa cells, and their oocytes require longer culture times and more complex IVM media than antral follicle oocytes [48–50]. The presence of LHR at this stage is important due to the role of LH in oocyte maturation and ovulation [51]. In this study, post-vitrification LHR expression was detected in granulosa and theca cells of secondary follicles, consistent with the findings of Yung *et al*. [47], although with less intensity than in antral follicles.

The LHR expression response in secondary follicles varied with PE concentration and vitrification duration. In the 0 μg/mL and 400 μg/mL PE groups, weak expression remained stable. The 200 μg/mL group showed a fluctuating pattern, from weak to negative (day 7) and then back to weak (day 14), indicating suboptimal stimulation. The 600 μg/mL PE group significantly increased expression (p < 0.05) from weak to moderate on day 7 and then slightly declined on day 14, although still higher than at day 0. This suggests an adaptive response to vitrification stress, where LHR expression increases after initial cell injury. The BPE 300 μg/mL group showed the highest expression on day 0, significantly different from other treatments (p < 0.05), but drastically declined and disappeared by days 7 and 14. This suggests that although the initial response was good, its effect did not persist under medium- to long-term vitrification.

The highest LHR expression in antral follicles was observed in the BPE 300 μg/mL group at day 14, followed by the PE 400 μg/mL group. This suggests that hormonal responsiveness increases in late follicular development, consistent with the role of LH in triggering luteinization and promoting oocyte maturation to metaphase II [38]. LHR expression in antral follicles confirms successful vitrification in maintaining follicular LH responsiveness.

LHR expression in antral follicles was observed across all treatment groups, ranging from weak to moderate, and was generally higher than that in earlier follicle stages. This agrees with the findings of Reyes *et al*. [52], who reported increased LHR gene and protein expression during follicular maturation in the estrous cycle. Expression dynamics varied across the treatments. In the EHS 0 μg/mL group, expression remained weak throughout vitrification, with a slight but statistically insignificant increase on day 7. In the 200 μg/mL group, expression decreased on day 7 and rebounded on day 14 but remained weak. Notably, the 400 μg/mL PE group showed a consistent increase from weak to moderate. Conversely, in the 600 μg/mL group, expression declined from moderate on day 0 to weak on day 14, suggesting a potential adverse effect of high dose over time. The BPE 300 μg/mL group had the highest overall expression scores; although expression dipped on day 7, it rebounded significantly on day 14 and remained in the moderate category.

In summary, PE concentrations of 400 and 600 μg/mL effectively increased LHR expression, particularly in primary and antral follicles, and maintained stability of expression during vitrification. The variability of responses across follicle stages and treatments underscores the importance of selecting the appropriate hormone type and dose to preserve follicular physiological integrity during and after cryopreservation. Thus, supplementing vitrification media with 400 and 600 μg/mL PE may enhance LHR expression across follicular stages in Aceh cattle ovaries up to 14-day post-vitrification. However, further studies are needed, as increased LHR expression does not always correlate positively with successful follicular development.

### Mechanistic pathways and hormonal interactions

Pituitary hormones, such as FSH and LH, are key components in maintaining follicular survival by modulating apoptosis through the PKA/Akt/forkhead box protein O1 (FoxO1) pathway. The anti-apoptotic effect of FSH on granulosa cells activates the PKA/Akt/FoxO1 signaling pathway. FSH promotes FoxO1 phosphorylation and cytoplasmic sequestration by activating the PKA and PI3K/Akt cascades. This prevents FoxO1 from translocating into the nucleus, where it would otherwise induce the transcription of pro-apoptotic genes such as Bim and FasL [29]. Consequently, the downregulation of these pro-apoptotic mediators promotes GC survival and follicular development. In the context of ovarian vitrification, this mechanism may explain the observed decrease in caspase-3 expression and the increased expression of FSHR in PE-treated ovarian tissues, suggesting that pituitary-derived gonadotropins may confer cytoprotective effects by modulating the FoxO1-mediated apoptotic pathway. A previous study by Kim and Li [53] has reported that FSH administration during vitrification resulted in a higher re-expansion rate compared with the control group. Similarly, LH supplementation during cryopreservation enhanced normal follicle production and upregulated the expression of VEGF, Cx37, and Cx43. In addition to FSH and LH, PE also contains growth hormone, which can reduce stromal apoptosis and fibrosis [54], indicating a potentially greater efficacy than FSH or LH alone.

### Limitations and future research

This study has several limitations that should be acknowledged. Although the hormonal quantification of bovine PE has now been added, other bioactive components that may influence follicular survival were not analyzed. Additionally, follicular viability was not directly assessed using methods such as trypan blue staining, TUNEL assay, or histomorphometric viability indices, which could have provided a more detailed evaluation of cellular integrity. The vitrification duration of 14 days represents a short-term storage period, and extrapolation of the findings to long-term cryostorage should therefore be performed with caution. Moreover, there was no correlation between receptor expression and oocyte maturation or fertilization potential, limiting the interpretation of receptor upregulation in terms of functional reproductive outcomes. To better understand the physiological relevance of PE supplementation in ovarian cryopreservation, future studies are recommended to examine whether receptor retention after vitrification correlates with subsequent oocyte maturation, fertilization, or early embryonic development.

### Practical implications

Therefore, incorporating PE into the vitrification medium could enhance post-thaw follicular viability and developmental competence in Aceh cattle, which are known for high genetic adaptability but low reproductive efficiency. This study introduces a novel application of bovine PE as a multi-hormonal supplement in vitrification media, potentially replacing exogenous gonadotropins. Unlike conventional single-hormone supplementation, PE provides a naturally balanced endocrine environment, which is hypothesized to enhance follicular viability and receptor expression in a species-specific manner. This factorial approach uniquely quantifies the concentration-dependent and temporal effects of PE supplementation on apoptosis and receptor integrity, thereby establishing a methodological model for optimizing cryopreservation media in indigenous cattle species. Beyond its laboratory relevance, this study advances a sustainable biotechnology approach consistent with One Health principles, utilizing abattoir-derived pituitary glands to produce functional cryoprotective agents for genetic conservation. This innovation reduces the dependency on imported reagents and aligns with circular bioresearch practices in low- to middle-income settings.

## CONCLUSION

This study demonstrated that supplementation of vitrification media with bovine PE significantly improved post-vitrification ovarian tissue quality in Aceh cattle. The results showed a clear reduction in caspase-3 expression, a key apoptotic marker, across all follicular stages, especially at 600 μg/mL PE after 7 days of vitrification, indicating decreased granulosa cell apoptosis and enhanced follicular survival. Concurrently, FSHR and LHR expression were upregulated in PE-treated groups, confirming that hormonal responsiveness and follicular functionality were preserved despite cryogenic stress. The interaction between PE concentration and vitrification duration revealed that moderate vitrification (7 days) combined with higher PE concentration yielded optimal receptor expression and the lowest apoptosis levels.

The study provides strong evidence that PE supplementation offers a multi-hormonal, synergistic advantage compared with single exogenous gonadotropins such as FSH or LH. The extract’s natural composition, including FSH, LH, and growth hormone, likely acts through multiple intracellular survival pathways, such as PKA–Akt–FoxO1, ERK1/2, and TGF-β/FoxL2, thereby promoting granulosa cell viability, inhibiting caspase-3 activation, and maintaining receptor integrity. These findings align with previous reports that gonadotropins reduce cryo-induced damage and support follicular development post-warming.

A major strength of this study lies in its comprehensive factorial design, integrating hormonal quantification, time-dependent vitrification effects, and receptor-specific immunohistochemical analyses. The combined evaluation of caspase-3, FSHR, and LHR provides a mechanistic understanding of how PE mediates follicular protection. Furthermore, the use of locally derived bovine PE underscores a sustainable and cost-effective biotechnological approach, reducing dependency on imported reagents while promoting the valorization of abattoir by-products for research and genetic conservation purposes.

In conclusion, bovine PE at 400–600 μg/mL effectively preserves follicular architecture, minimizes apoptosis, and sustains gonadotropin receptor expression during ovarian vitrification of Aceh cattle. These findings establish a foundation for developing economical, species-specific cryopreservation protocols to safeguard the genetic resources of indigenous livestock. Future research should extend this approach to evaluate long-term follicular viability, oocyte maturation, and embryo development following thawing, thereby bridging molecular findings with functional reproductive outcomes.

## AUTHORS’ CONTRIBUTIONS

CIN and TNS: Designed the study, performed data analysis, and drafted the manuscript. CIN: Collected samples. SW: Performed IHC analysis. NWKK: Supervised the field and laboratory work. AS: Data analysis and drafted and revised the manuscript. All authors have read and approved the final version of the manuscript.
